# PMC-12, a Prescription of Traditional Korean Medicine, Improves Amyloid **β**-Induced Cognitive Deficits through Modulation of Neuroinflammation

**DOI:** 10.1155/2015/768049

**Published:** 2015-04-07

**Authors:** Min Young Park, Yeon Suk Jung, Jung Hwa Park, Young Whan Choi, Jaewon Lee, Cheol Min Kim, Jin Ung Baek, Byung Tae Choi, Hwa Kyoung Shin

**Affiliations:** ^1^Division of Meridian and Structural Medicine, School of Korean Medicine, Pusan National University, Yangsan, Gyeongnam 626-870, Republic of Korea; ^2^Korean Medical Science Research Center for Healthy Aging, Pusan National University, Yangsan, Gyeongnam 626-870, Republic of Korea; ^3^Department of Horticultural Bioscience, College of Natural Resource and Life Science, Pusan National University, Miryang, Gyeongnam 626-706, Republic of Korea; ^4^Department of Pharmacy, College of Pharmacy, Pusan National University, Busan 609-735, Republic of Korea; ^5^Research Center for Anti-Aging Technology Development, Pusan National University, Busan 609-735, Republic of Korea; ^6^Division of Humanities and Social Medicine, School of Korean Medicine, Pusan National University, Yangsan, Gyeongnam 626-870, Republic of Korea

## Abstract

PMC-12 is a prescription used in traditional Korean medicine that consists of a mixture of four herbal medicines, *Polygonum multiflorum*, *Rehmannia glutinosa*, *Polygala tenuifolia*, and *Acorus gramineus*, which have been reported to have various pharmacological effects on age-related neurological diseases. In the present study, we investigated whether PMC-12 improves cognitive deficits associated with decreased neuroinflammation in an amyloid-*β*-(A*β*-) induced mouse model and exerts the antineuroinflammatory effects in lipopolysaccharide-(LPS-) stimulated murine BV2 microglia. Intracerebroventricular injection of A*β*_25−35_ in mice resulted in impairment in learning and spatial memory, whereas this was reversed by oral administration of PMC-12 (100 and 500 mg/kg/day) in dose-dependent manners. Moreover, PMC-12 reduced the increase of A*β* expression and activation of microglia and astrocytes in the A*β*_25−35_-injected brain. Furthermore, quantitative PCR data showed that inflammatory mediators were significantly decreased by administration of PMC-12 in A*β*-injected brains. Consistent with the *in vivo* data, PMC-12 significantly reduced the inflammatory mediators in LPS-stimulated BV2 cells without cell toxicity. Moreover, PMC-12 exhibited anti-inflammatory properties via downregulation of ERK, JNK, and p38 MAPK pathways. These findings suggest that the protective effects of PMC-12 may be mediated by its antineuroinflammatory activities, resulting in the attenuation of memory impairment; accordingly, PMC-12 may be useful in the prevention and treatment of AD.

## 1. Introduction

Alzheimer's disease (AD), the most common cause of progressive cognitive impairment in the elderly, is pathologically characterized by the deposition of senile plaques composed of amyloid-*β* (A*β*) and neurofibrillary tangles in vulnerable brain regions. Increased A*β* deposition is believed to play an important role in AD pathogenesis [1]. Numerous studies have suggested that neuroinflammation is involved in the mechanism of AD pathogenesis [2] and the accumulation of A*β* plaques in the brains plays an important role in initiating neuroinflammatory and neurotoxic responses that result in further cell damage in AD patients [3–5]. It has also been reported that nonsteroidal anti-inflammatory drug (NSAID) treatment delays AD onset, acts to ameliorate symptomatic severity, and slows AD progression [6, 7]. However, NSAIDs could have potentially serious side effects in high doses or when used over the long term. Therefore, traditional herbal medicines or natural products have attracted attention as alternative or complementary approaches for AD treatment because they have fewer side effects [8].

PMC-12 is a multiherb mixture of* Polygonum multiflorum*,* Rehmannia glutinosa*,* Polygala tenuifolia*, and* Acorus gramineus*. Each of these herbs has been used extensively in prescriptions for age-related neurological diseases including AD [9–11]. These herbal medicines have been shown to express various pharmacological activities against age-related brain disease, such as neuroprotective, antioxidant, and anti-inflammatory effects. Among PMC-12,* Polygonum multiflorum* is known to have antiapoptotic, anti-inflammatory, and antioxidative effects, and it is also known to reduce blood cholesterol and improve hair growth and learning and memory [12–15]. Additionally, a primary bioactive constituent, stilbene glucoside (2,3,5,4′-tetrahydroxystilbene-2-*O*-*β*-*D*-glucoside), has been reported to possess antioxidative, anti-inflammatory, and antiapoptotic effects, and it has also been reported to improve memory and learning ability [16–21]. In addition,* Rehmannia glutinosa, Polygala tenuifolia*,* Acorus gramineus*, and their major effective compounds, catalpol, 3′6-disinapoyl sucrose, and asarone, have been reported to improve learning and memory, and they have also been reported to exert neuroprotective and anti-inflammatory effects [22–29]. Taken together, the neuroprotective and antiaging effects of these four herbal medicines indicate that PMC-12 may have potential for treatment of AD.

Based on accumulating evidence of the pathological roles of A*β* in the progress of AD, A*β*_25–35_-injected mice have become a useful animal model of AD for evaluation of anti-AD drugs [30]. To evaluate the therapeutic potential of PMC-12 for the treatment of AD, we tested the cognitive enhancing effect of PMC-12 using this model. Specifically, A*β*-induced pathological and inflammatory alterations were examined by immunohistochemistry and real-time PCR and the learning and memory deficits were evaluated by a water maze. We also conducted an* in vitro* study using BV2 microglia cells to elucidate the mechanism of cognitive enhancement of PMC-12.

## 2. Methods

### 2.1. Preparation of PMC-12 Extract

The dried roots of* Polygonum multiflorum*,* Rehmannia glutinosa*,* Polygala tenuifolia*, and* Acorus gramineus* were purchased from Hwalim Natural Drug (Busan, Korea) and a voucher specimen (accession number PMC-12) was deposited at the Department of Korean Medical Science, Pusan National University (Yangsan, Korea). Dried powdered* P. multiflorum* (25.5 kg),* R. glutinosa* (9.5 kg),* P. tenuifolia* (7.5 kg), and* A. gramineus* roots (7.5 kg) were immersed in 450 L of distilled water (DW) and boiled at 115 ± 5°C for 150 min. The resultant extract was centrifuged (2000 ×g for 20 min at 4°C) and then filtered through a 0.2-*μ*m filter. The filtrate was subsequently concentrated in vacuo at 70 ± 5°C under reduced pressure, after which it was converted into a fine spray-dried powder at a yield rate of 4.6% (2.3 kg) in a vacuum drying apparatus. Finally, the solid form of the spray-dried extract was dissolved with dimethyl sulfoxide (DMSO) for use as PMC-12 in experiments.

For analysis of quantity for PMC-12, sample of 0.5 g dry weight was sonicated in 10 mL MeOH, filtered through a 0.45 *μ*m membrane filter before HPLC analysis. HPLC using G1100 systems (Agilent Technologies, Waldbronn, Germany) was performed on a Luna C_18_ column (5 *μ*m, 150 mm × 3.0 mm i.d., Phenomenex, Torrance, CA, USA) with a mobile phase gradient of acetonitrile–water (0 to 100) for 35 min. The injection volume was 10 *μ*L of sample and mobile phase flow rate 0.4 mL/min with UV detection at 254 nm for 2,3,5,4′-tetrahydroxystilbene-2-O-*β*-D-glucoside (THS) and 3′,6-disinapoyl sucrose (DISS) and at 203 nm for catalpol. Acquisition and analysis of chromatographic data were performed using Agilent chromatographic Workstation software (Agilent Technologies). Stock solutions of THS, DISS, and catalpol were prepared for quantification of PMC-12. The contents of PMC-12 were determined by regression equations, calculated in the form of *y* = *ax* + *b*, where *x* and *y* were peak area and contents of the compound. The limits of detection (LOD) and the limits of quantification (LOQ) under the current chromatographic conditions were determined at a signal-to-noise ratio of 3 and 10, respectively.

### 2.2. A*β*_25–35_ Injection Model

To evaluate the effects of PMC-12 on A*β*-induced cognitive impairment, a mouse model produced using a modified version of previously reported method [31] was used. Male C57BL/6J mice (20–25 g) were housed under diurnal lighting conditions and allowed food and tap water* ad libitum*. The animal protocol used in this study was approved by the Pusan National University-Institutional Animal Care and Use Committee (PNU-IACUC) for ethical procedures and scientific care (Approval no. PNU-2013-0380). C57BL/6J mice were randomly assigned to four groups, saline-injected normal control, A*β*_25–35_-injected DW-treated vehicle, A*β*_25–35_-injected PMC-12-treated (100 mg/kg), and A*β*_25–35_-injected PMC-12-treated (500 mg/kg). Anesthesia was achieved by isoflurane (2% induction and 1.5% maintenance, in 70% N_2_O and 30% O_2_) administered via a face mask. The depth of anesthesia was checked by the absence of cardiovascular changes in response to a tail pinch. Rectal temperature was maintained at 36.5°C–37.5°C using a Panlab thermostatically controlled heating mat (Harvard Apparatus, Holliston, MA). A*β*_25–35_ (Sigma-Aldrich, St. Louis, MO) was dissolved in saline and incubated at 37°C for 4 days to form aggregated A*β* before use. Aggregated A*β* solution (10 nmol in 5 *μ*L of saline) was injected intracerebroventricularly (icv) into the mice 1 mm lateral to the midline, 0.5 mm posterior to the bregma, and 3 mm deep using a 25 *μ*L Hamilton syringe with a 26-gauge needle (Hamilton, Reno, NV) at a rate of 0.5 *μ*L/min using a stereotaxic injector (KD Scientific, Holliston, MA). The vehicle group of mice received icv injections of an equal volume of saline. Either PMC-12 (100 or 500 mg/kg) or an equal volume of distilled water (vehicle) was administered orally and daily for 3 weeks after A*β*_25–35_ injection.

### 2.3. Morris Water Maze Task

Spatial learning and memory deficits were assessed using the Morris water maze task as previously described [31] with minor modification. The experiment was performed on mice 3 weeks after A*β*_25–35_ injection. The maze consisted of a 1.15 m diameter pool painted flat white. A 10 cm diameter platform was placed halfway between the center of the pool and the edge and was positioned 1 cm below the surface of the water. The water in the pool was made opaque by the addition of powdered milk. The water temperature was 19°C–21°C. The water tank was located in a test room containing many cues external to the maze that remained unchanged throughout the water maze task. Each mouse was subjected to a series of three trials per day for 4 days. For each trial, mice were randomized to one of four directional starting locations (north, south, east, and west) and then placed in the pool facing the wall. When a mouse located the platform, it was permitted to remain on it for 3 sec. Mice were given a maximum of 90 sec to find the submerged platform. Swimming was video tracked, and latency and path length from the platform were analyzed using Smart software (Panlab, Barcelona, Spain).

### 2.4. Immunohistochemistry

Three weeks after A*β*_25–35_ injection, mice were deeply anesthetized with thiopental sodium and subsequently perfused transcardially with cold PBS followed by 4% paraformaldehyde for fixation. The brain of each mouse was then removed and further fixed for 24 h in 4% paraformaldehyde at 4°C followed by cryoprotection in 20% sucrose for 72 h at 4°C. Next, the isolated brains were frozen in an optical cutting temperature medium for frozen tissue specimens (Sakura Finetek, Torrance, CA) and then stored at −80°C until examined. The frozen brains were cut at a thickness of 14 *μ*m using a CM 3050 cryostat (Leica Microsystems, Wetzlar, Germany), after which the sections were immunostained with antibodies against A*β* (4G8, Covance, Emeryville, CA), Iba-1 (Wako Pure Chemical Industries, Osaka, Japan), and GFAP (Dako, Glostrup, Denmark) at 4°C overnight. After additional incubation with biotinylated secondary antibody, the samples were incubated in ABC reagent (Vector Laboratories, Burlingame, CA). Reactions were then visualized by development in 3,3′-diaminobenzidine substrates (Vector Laboratories). All samples were visualized using a light microscope (Carl Zeiss, Jena, Germany).

### 2.5. Cell Culture

BV2 cells (murine microglia) were cultured in Dulbecco's Modified Eagle's Medium (DMEM, Gibco, Carlsbad, CA) with 10% fetal bovine serum (HyClone, Logan, UT), 100 U/mL penicillin, and 100 *μ*g/mL streptomycin in a humidified atmosphere containing 5% CO_2_ in air at 37°C. PMC-12 was dissolved in DMSO, after which dilutions were made in DMEM. The final concentration of DMSO in the medium was less than 0.01% (vol/vol) which showed no influence on cell growth. In all experiments, cells were pretreated with the indicated concentrations of PMC-12 for 1 h before LPS (1 *μ*g/mL) treatment for 24 h.

### 2.6. Western Blotting

Proteins from BV2 cells were isolated according to standard techniques, separated by 8% sodium dodecyl sulfate-polyacrylamide gel electrophoresis (SDS-PAGE), and transferred onto a polyvinylidene fluoride membrane (PVDF, Millipore, Bedford, MA). Blots were then probed for anti-COX2 (Santa Cruz Biotechnology, Dallas, TX), anti-iNOS (BD Biosciences, San Jose, CA), anti-p38, anti-p-p38, anti-JNK, anti-p-JNK, anti-ERK, or anti-p-ERK (Cell Signaling, Danvers, MA), after which they were incubated with secondary antibody conjugated with horseradish peroxidase. The intensity of chemiluminescence was measured using an ImageQuant LAS 4000 apparatus (GE Healthcare Life Sciences, Buckinghamshire, UK). The membrane was then reprobed with an anti-*β*-actin antibody (Sigma-Aldrich) as an internal control.

### 2.7. Reverse Transcription-Polymerase Chain Reaction (RT-PCR) and Real-Time PCR

Total RNA was isolated from BV2 cells or mouse brains using Trizol (Invitrogen, Carlsbad, CA) according to the manufacturer's recommendations. The RNA was then reverse-transcribed for one hour at 42°C with Moloney Murine Leukemia Virus reverse transcriptase (Promega, Madison, WI) to produce cDNA. For RT-PCR, RT-generated cDNA encoding the iNOS, COX-2, and GAPDH genes was amplified by PCR using the primers shown in Table 1. Products were then size-separated by electrophoresis on 2% agarose gels and visualized after staining with ethidium bromide. The following PCR conditions were applied: iNOS: 35 cycles of denaturation at 94°C for 30 s, annealing at 60°C for 30 s and extension at 72°C for 30 s; COX-2: 28 cycles of denaturation at 94°C for 30 s, annealing at 60°C for 30 s and extension at 72°C for 30 s; GAPDH: 25 cycles of denaturation at 94°C for 30 s, annealing at 55°C for 30 s and extension at 72°C for 30 s. Real-time PCR was conducted using a Rotor-Gene Q real-time PCR system (Qiagen, Hilden, Germany) with SYBR Green PCR Master Mix (Qiagen), and the results were normalized to GAPDH gene expression. All experiments were performed in triplicate and repeated at least three times using the primers shown in Table 1. The threshold cycles (Ct) were used to quantify the mRNA expression of target genes.

### 2.8. Data Analysis

The data are expressed as the means ± SEM. Statistical comparisons were performed using a paired or unpaired Student's *t*-test and one-way analysis of variance (ANOVA) or two-way ANOVA for repeated measures followed by Fisher's protected least significant difference test. *P* < 0.05 was considered statistically significant.

## 3. Results

### 3.1. PMC-12 Improves Cognitive Impairment in A*β*-Injected Mice

**Figure 1:**
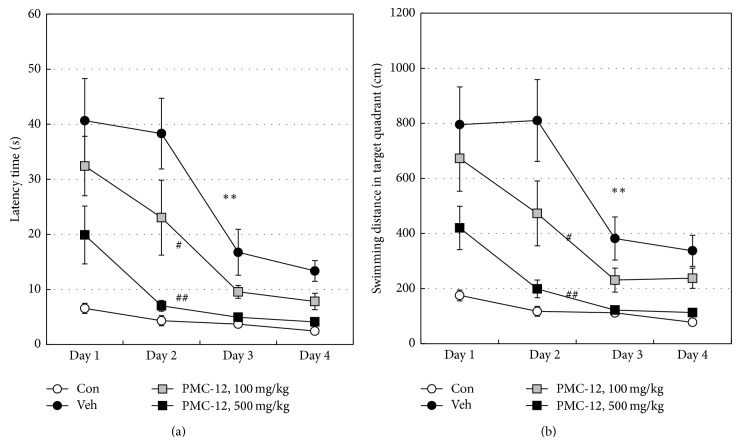
Effects of PMC-12 on spatial learning and memory in A*β*_25–35_-injected mice based on the Morris water maze test. Changes in escape latency (a) and swimming distance (b) to reach the platform in mice treated with distilled water (Veh) or PMC-12 (100 or 500 mg/kg/day, orally for 3 weeks) at 3 weeks after A*β*_25–35_ injection. A*β*_25–35_ injection significantly increased escape latency time and swimming distance (*P* < 0.01 versus control, two-way ANOVA), which was reversed by PMC-12 (100 and 500 mg/kg, *P* < 0.05 and *P* < 0.01 versus vehicle, resp., two-way ANOVA). ^**^ *P* < 0.01 versus control (Con); ^#^ *P* < 0.05 and ^##^ *P* < 0.01 versus vehicle (Veh). Data shown are the mean ± SEM from nine separate experiments.

PMC-12 was standardized based on the THS, catalpol, and DISS, which were reported to be the major bioactive constituents of* Polygonum multiflorum*,* Rehmannia glutinosa*, and* Polygala tenuifolia*. Based on UV maximal absorption, we detected THS and DISS at 254 nm and catalpol at 203 nm for quantitative analysis. The contents of THS, catalpol, and DISS in PMC-12 were 3.085 ± 0.271%, 0.785 ± 0.059%, and 0.352 ± 0.058%, respectively. Linear calibration curve showed good linear regression (*r* ^2^ > 0.999) within test ranges; the LOD (*S*/*N* = 3) and the LOQ (*S*/*N* = 10) were less than 1.5 and 4.5 *μ*g at 254 nm for THS and DISS and at 203 nm for catalpol. To determine if PMC-12 reversed A*β*-induced memory deficit, we evaluated memory performance using the Morris water maze test in A*β*_25–35_-injected mice. Oral administration of PMC-12 (100 and 500 mg/kg/day) for 3 weeks had no effect on body weight (data not shown). As shown in Figure 1, icv injection of A*β*_25–35_ resulted in a significantly increased escape latency time and swimming distance in the target quadrant compared to the control group at 3 weeks after A*β* injection (*P* < 0.01 versus control). Treatment of mice with PMC-12 (100 and 500 mg/kg) led to significant attenuation of the increase in escape latency time and swimming distance in a dose-dependent manner compared to vehicle (100 and 500 mg/kg, *P* < 0.05 and *P* < 0.01 versus vehicle, resp.).

### 3.2. PMC-12 Attenuates Neuroinflammatory Responses in A*β*-Injected Mice

**Figure 2:**
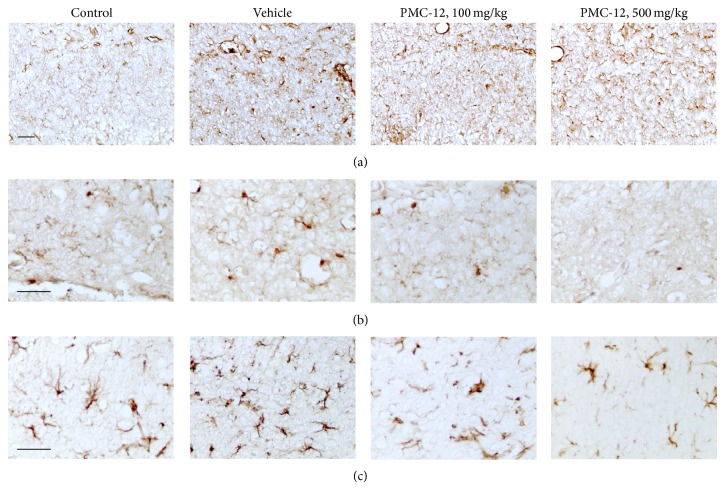
Effects of PMC-12 on A*β* expression and microglia and astrocyte activations in A*β*_25–35_-injected mouse brain. Photomicrographs showed immunohistochemically (a) A*β* (4G8), (b) Iba-1 (a sensitive marker of microglial activation), and (c) GFAP (a sensitive marker of astrocyte activation) stained brains at 3 weeks after A*β*_25–35_ injection. Either PMC-12 (100 and 200 mg/kg) or an equal volume of distilled water (Veh) was administered once a day for 3 weeks. A*β*, Iba-1, and GFAP immunoreactivities were increased in the CA1 regions of the hippocampus with vehicle treatment in mice but were attenuated by treatment with PMC-12. The results shown are representative of those obtained from four independent experiments. The scale bar is 20 *μ*m.

**Figure 3:**
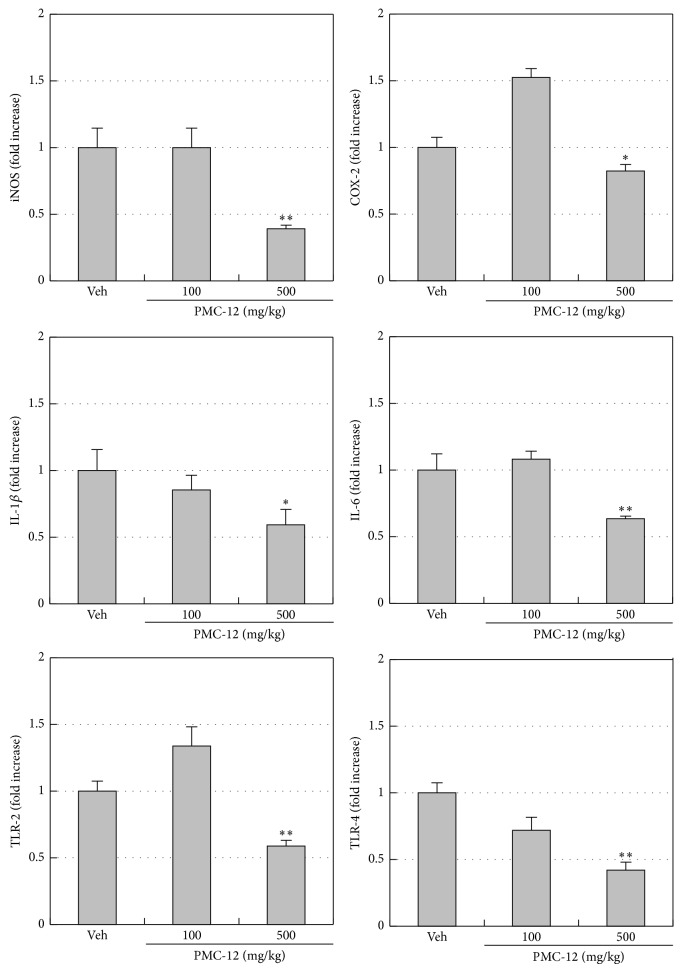
Effects of PMC-12 on inflammatory mediators in A*β*_25–35_-injected mouse brain. The brain tissues of A*β*_25–35_-injected mice were analyzed for iNOS, COX-2, IL-1*β*, IL-6, TLR-2, and TLR-4 mRNA levels by real-time PCR. These gene expression levels were normalized to GAPDH. Either PMC-12 (100 and 500 mg/kg) or an equal volume of distilled water (Veh) was administered orally 3 weeks after A*β*_25–35_ injection. PMC-12 significantly decreased iNOS, COX-2, IL-1*β*, IL-6, TLR-2, and TLR-4 mRNA levels in A*β*-injected brain. ^*^ *P* < 0.05 and ^**^ *P* < 0.01 versus vehicle (Veh). Data shown are the mean ± SEM of values from four separate experiments.

Next, we identified whether PMC-12 prevents the accumulation of A*β* in the brains of mice subjected to icv injection of A*β*_25–35_. Elevated A*β* immunoreactivity in the CA1 region of the hippocampus of the A*β*-injected mouse brain was observed and the increase of A*β* immunoreactivity was markedly decreased by the administration of PMC-12 (Figure 2). Iba-1 is a sensitive marker of microglial activation and GFAP is a sensitive marker of astrocyte activation. Numerous Iba-1 immunostained microglia and GFAP immunostained astrocytes were evident in the hippocampus of mice receiving icv injections of A*β*_25–35_ and treatment with vehicle. However, administration of PMC-12 at 100 and 500 mg/kg for 3 weeks after surgery prevented the A*β*-induced increase of Iba-1 and GFAP immunoreactivity, with 500 mg/kg PMC-12 leading to almost complete attenuation of Iba-1 expression (Figure 2). Next, we identified the inflammatory mediators in A*β*-injected mouse brains using real-time PCR analysis (Figure 3). Treatment of 500 mg/kg PMC-12 significantly decreased iNOS, COX-2, IL-1*β*, IL-6, TLR-2, and TLR-4 in A*β*-injected brains (Figure 3).

### 3.3. Effect of PMC-12 on LPS-Induced Expression of Protein and mRNA for Inflammatory Mediators in BV2 Microglia Cells

**Figure 4:**
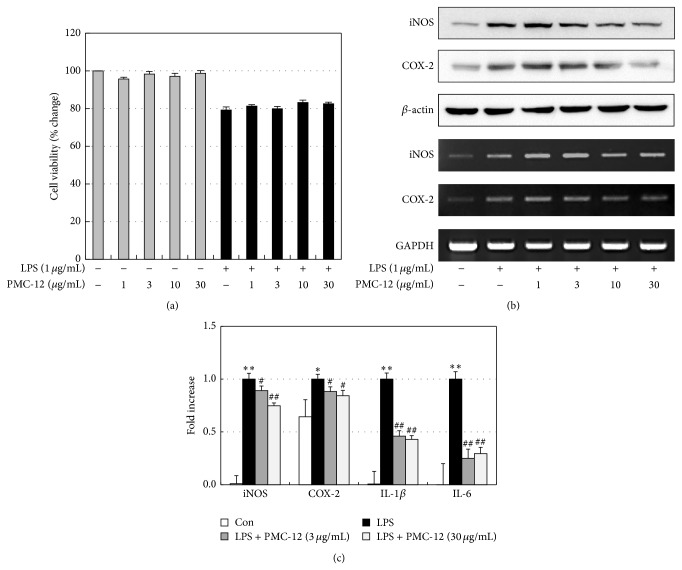
Effects of PMC-12 on cell viability and LPS-induced inflammatory mediators in BV2 microglia cells. Cells were treated with the indicated concentrations of PMC-12 (1, 3, 10, or 30 *μ*g/mL) 1 h before LPS (1 *μ*g/mL) treatment for 24 h (MTT reduction assays and Western blot analysis) or 6 h (RT-PCR and real-time PCR). (a) Cell viability was assessed by MTT reduction assays and the results were expressed as the percentage of surviving cells over control cells (no addition of LPS and PMC-12). (b) The expressions of iNOS and COX-2 protein and mRNA were determined by Western blot analysis and RT-PCR, respectively. (c) These data were confirmed by real-time PCR that showed PMC-12 significantly reduced the inflammatory mediators iNOS, COX-2, IL-1*β*, and IL-6 levels in LPS-stimulated BV2 cells. ^**^ *P* < 0.01 versus cells without LPS (Con, control); ^#^ *P* < 0.05 and ^##^ *P* < 0.01 versus cells treated with LPS in the absence of PMC-12. Data shown are the means ± SEM of values from four separate experiments.

Cell viability was assessed by MTT reduction assays (Figure 4(a)). PMC-12 treatment with or without LPS did not have a significant effect on cell viability. The inhibitory effects of PMC-12 on the expression of iNOS and COX-2 protein and mRNA were determined by Western blot analysis and RT-PCR, respectively. Levels of iNOS and COX-2 proteins were markedly upregulated 24 h after LPS (1 *μ*g/mL) treatment, and PMC-12 attenuated iNOS and COX-2 protein expression in LPS-stimulated BV2 microglia in a concentration-dependent manner (Figure 4(b)). The effects of PMC-12 on iNOS and COX-2 mRNA levels were also evaluated 6 h after LPS treatment (Figure 4(b)). RT-PCR analysis showed that the reduction in iNOS and COX-2 mRNA was correlated with the reduction in the corresponding protein levels. These data were confirmed by real-time PCR, which showed that PMC-12 significantly reduced the inflammatory mediators iNOS, COX-2, IL-1*β*, and IL-6 in LPS-stimulated BV2 cells (Figure 4(c)).

### 3.4. Effect of PMC-12 on LPS-Induced MAPKs Activation in BV2 Microglia Cells

**Figure 5:**
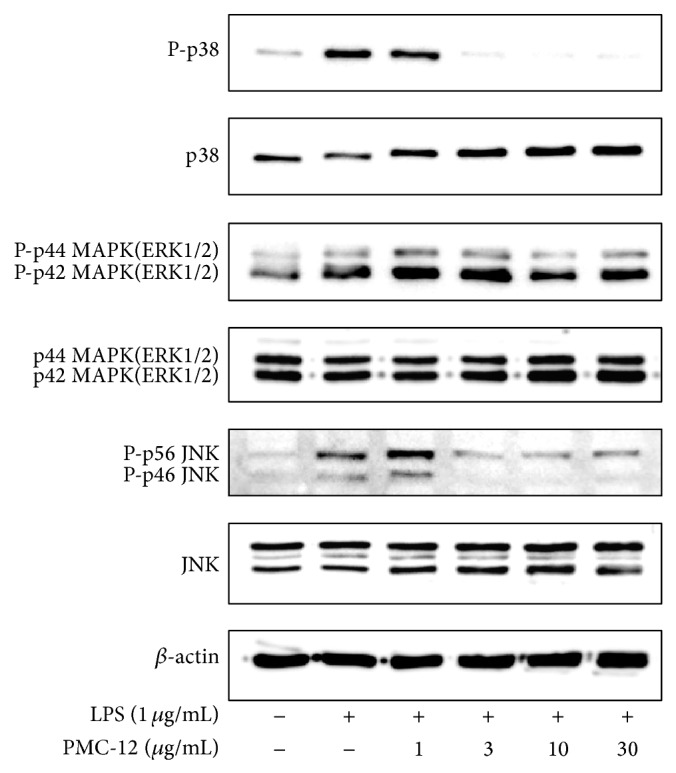
Effects of PMC-12 on MAPKs activation induced by LPS in microglia. BV2 cells were treated with the indicated dose of PMC-12 (1, 3, 10, or 30 *μ*g/mL) 1 h before LPS treatment (1 *μ*g/mL) for 24 h. Total protein (50 *μ*g) was subjected to 8% SDS-PAGE, followed by Western blotting using anti-p38, anti-ERK-1/2, and anti-JNK. PMC-12 significantly inhibited p38, ERK-1/2, and JNK MAPK activation in a dose-dependent manner. Results are representative of those obtained from four independent experiments. Actin was used as an internal control.

Mitogen-activated protein kinases are the most important signaling molecules involved in activated microglia [32]. Therefore, we investigated the effects of PMC-12 on the activation of p38, ERK-1/2, and JNK 24 h after LPS stimulation of BV2 cells. Phosphorylation of p38, ERK, and JNK was markedly upregulated in response to LPS stimulation, whereas treatment of the cells with PMC-12 significantly inhibited p38, ERK-1/2, and JNK MAPK activation in a dose-dependent manner (Figure 5). These results suggest that PMC-12 is capable of disrupting key signal transduction pathways activated by LPS in BV2 microglia.

## 4. Discussion

Here, we report the effects of PMC-12 on the prevention of A*β*-induced cognitive deficits, neuropathological changes, and neuroinflammatory responses in a mouse model and LPS-stimulated murine BV2 microglia cells. Intracerebroventricular injection of A*β*_25–35_ led to impairment in learning and spatial memory function as well as increases in A*β*, Iba-1, and GFAP immunoreactivity. PMC-12 treatment prevented A*β*-induced increases in both AD-related neuropathological markers (A*β*, Iba-1, and GFAP) and inflammatory mediators (iNOS, COX-2, IL-1*β*, IL-6, TLR-2, and TLR-4) as well as in learning and spatial memory deficits in A*β*-treated mice. In addition, this study revealed that the antineuroinflammatory actions of PMC-12 are associated with prevention of MAPK activation. Taken together, these findings indicate that the cognitive-enhancing activity of PMC-12 might result, in part, from inhibition on the accumulation of A*β* and the reduction of neuroinflammation. These findings suggest that PMC-12 has the potential for use in the prevention or treatment of AD.

There is still no animal model that can mimic all of the cognitive, biochemical, and neuropathological abnormalities observed in AD patients. A*β*_25–35_, which is the core fragment of full-length A*β*_1–42_, exerts neurotoxic effects including memory deficits, cholinergic dysfunction, neuronal apoptosis, and oxidative stress [30]. We also demonstrated that both AD-like and inflammatory pathologies were observed following icv injection of 10 nmol A*β*_25–35_ into the mice [33]. Therefore, we used icv injection of A*β*_25–35_ in C57BL/6 mice as the animal model of AD for evaluation of anti-AD drugs. Learning and memory deficits are early and critical symptoms of AD [34]. The cognitive-enhancing activity of PMC-12 was evaluated by the Morris water maze test, which is one of the most frequently used laboratory tools in spatial learning-memory performance [31]. The results showed a significant increase in learning and spatial memory deficits in mice receiving icv A*β*_25–35_ injections relative to sham treated mice. In accordance with the water maze results, we observed elevated accumulation of extracellular A*β* in the hippocampus of A*β*-injected mouse brains. The anti-A*β* antibody (clone: 4G8) used in this study recognizes the 17–24 residues of A*β* peptide which is different from the A*β*_25–35_ domain we injected. Therefore, the immunoreactivity to A*β* in the hippocampus seems like a result of endogenous expressed A*β* rather than the immune-detected A*β* by icv injection of A*β*_25–35_. This is in agreement with previous studies that demonstrated that administration of A*β*_25–35_ could upregulate amyloid precursor protein (APP) and endogenous A*β* production [35]. Thus, the learning and spatial memory deficits are consistent with the immunohistochemical findings. Oral administration of PMC-12 (100 and 500 mg/kg/day) for 3 weeks improved cognitive function and reduced the A*β*_25–35_-induced increase of A*β* immunoreactivity in a dose-dependent manner. In addition, the spatial learning-memory performance of mice receiving 500 mg/kg PMC-12 recovered to almost the control level. In contrast, the swim speed was not altered by PMC-12 (data not shown), indicating that the changes in escape latency were independent of general motor activity. Thus, the results from both the immunohistochemical data and behavior testing suggest that PMC-12 has the potential for use as an anti-AD drug.

Inflammation plays an important role in memory loss associated with AD and activation of microglia is a key component of the inflammatory response in AD [2, 36]. Depending on the mode of activation, there are two types of microglia: neurotoxic microglia and neuroprotective microglia [37]. In AD, A*β* aggregates activate neurotoxic microglia that secrete inflammatory mediators such as TNF-*α*, IL-1*β*, and IL-6 to coactivate astrocytes and to induce neuronal death, which in turn will amplify microglia. On the other hand, protective microglia mediate A*β* clearance and removal of cell debris and promote neuroregeneration. Therefore, regulation of the microglial role may be a useful therapeutic strategy for AD. In this study, prominent Iba-1 immunostained microglia and GFAP immunostained astrocytes were observed in the CA1 region of the hippocampus in A*β*-treated mice relative to sham treated mice. In addition, PMC-12 treatment (100 and 500 mg/kg/day) for 3 weeks prevented the A*β*-induced increase of Iba-1 and GFAP immunoreactivity, with 500 mg/kg PMC-12 almost completely attenuating Iba-1 expression. These findings suggest that the decrease of neuroinflammation and improvement of cognitive function are likely a result of inhibitory effect of PMC-12 on neurotoxic microglia activation. Regarding the inhibitory effect of PMC-12 on A*β* production, other mechanisms might be involved and further studies should be conducted.

Many studies have suggested that the expression of inflammatory cytokines is upregulated in A*β*-induced or scopolamine-induced AD models and that these cytokines may play a role in several events in the pathological cascade of AD [38, 39]. These results suggest that inflammatory reactions are related to the cognitive and functional decline that occurs in AD. Based on these findings, many anti-inflammatory drugs have been tested for their abilities to delay neuronal death, including cyclooxygenase-2 inhibitors [40, 41] and inducible nitric oxide synthase inhibitors [42]. In the present study, PMC-12 administration caused a decrease in inflammatory mediators (iNOS, COX-2, IL-1*β*, IL-6, TLR-2, and TLR-4) as well as learning and memory deficits in the A*β*-injected brain. We also evaluated the anti-inflammatory effects of PMC-12 in the system by measuring nitric oxide (NO) and prostaglandin E_2_ (PGE_2_) levels in the plasma of icv A*β*_25–35_-injected mice (data not shown). A*β*_25–35_ injection resulted in a marked increase in PGE_2_ release but not NO production relative to the control. However, PMC-12 inhibited this PGE_2_ plasma level in a concentration-dependent manner, with 500 mg/kg PMC-12 leading to a decrease to almost the control level. These data suggest that the cognitive-enhancing activity of PMC-12 is involved in modulating inflammation.

Activation of microglia is a key component of the inflammatory response in AD [36]. Activation of microglia results in the release of inflammatory mediators including NO, PGE_2_, reactive oxygen species, and proinflammatory cytokines, such as IL-1*β*, IL-6, and TNF-*α* [43]. Therefore, we evaluated the effects and mechanism of PMC-12 on production of LPS-stimulated proinflammatory mediators in BV2 microglia. Consistent with the* in vivo* data, PMC-12 significantly reduced iNOS, COX-2, IL-1*β*, and IL-6 in LPS-stimulated BV2 cells without cell toxicity. Various intracellular signaling pathways such as MAPKs are involved in inflammatory mediator expression [32]. Activation of MAPKs such as p38, ERK1/2, and JNK regulated the expression of inflammatory genes including IL-1*β*, iNOS, and COX-2. Therefore, we investigated the effects of PMC-12 on LPS-stimulated phosphorylation of p38, ERK-1/2, and JNK in BV2 microglia. Our results indicated that PMC-12 is a potent inhibitor of activation of MAPKs induced by LPS stimulation in BV2 microglia, suggesting that COX-2, iNOS, IL-1*β*, and IL-6 inhibition by PMC-12 in LPS-stimulated BV2 microglia might be due to its inhibitory effects on the MAPKs signaling pathway.

In conclusion, the results of this study suggest that PMC-12 markedly improves A*β*_25–35_-induced cognitive deficits and that these effects are mediated by the antineuroinflammatory properties of PMC-12. Other mechanisms of action were not investigated; however, a neuroprotective effect might also be a major mechanism because each component herb of PMC-12 has been reported to exert neuroprotective effects. Although the water extract of PMC-12 contains many bioactive compounds, it is not known which compounds are responsible for its antineuroinflammatory effects. Future studies should be conducted to identify the compounds from PMC-12 which are responsible for protecting cognitive impairment. Overall, the results of this study indicate that PMC-12 has the potential to reduce cognitive and neuropathological deficits and may represent a new approach for AD treatment.

## Figures and Tables

**Table 1:** Sequences of primers used in RT-PCR and real-time PCR analysis.

Gene	Primer	Length	Sequence
RT-PCR			
Mouse iNOS	Sense primer Antisense primer	461	CACTTGGATCAGGAACCTGAAG CCAGCTTCTTCAATGTGGTAGC
Mouse COX-2	Sense primer Antisense primer	271	TTCAACACACTCTATCAC AGAAGCGTTTGCGGTACT
Mouse GAPDH	Sense primer Antisense primer	486	ATGACCACAGTCCATGCCATCA TTACTCCTTGGAGGCCATGTAG
Real-time PCR			
Mouse iNOS	Sense primer Antisense primer	93	TCCTGGACATTACGACCCCT AGGCCTCCAATCTCTGCCTA
Mouse COX-2	Sense primer Antisense primer	185	AGAAACGGCTACCACATCCAA GGGTCGGGAGTGGGTAATTT
Mouse IL-1*β *	Sense primer Antisense primer	196	GGATGAGGACATGAGCACCT TCCATTGAGGTGGAGAGCTT
Mouse IL-6	Sense primer Antisense primer	191	AGTTGCCTTCTTGGGACTGA CAGAATTGCCATTGCACAAC
Mouse GAPDH	Sense primer Antisense primer	81	ACTGAGCAAGAGAGGCCCTA TTATGGGGGTCTGGGATGGA
